# Beta-blockade in experimental fluid-resuscitated sepsis: acute haemodynamic effects of esmolol differ in predicted survivors and non-survivors

**DOI:** 10.1186/2197-425X-3-S1-A801

**Published:** 2015-10-01

**Authors:** W Khaliq, DT Andreis, M Singer

**Affiliations:** University College London, Bloomsbury Institute of Intensive Care Medicine, London, United Kingdom; Università degli Studi di Milano, Dipartimento di Fisiopatologia Medico-Chirurgica e dei Trapianti, Milan, Italy

## Introduction

Beta-blockade therapy during sepsis has a sound rationale in view of its cardiac, metabolic, inflammatory and other effects [1]. Whether it is safe and efficacious in both good prognosis and poor prognosis patients is yet to be ascertained. We have developed a 72-h fluid-resuscitated rat model of faecal peritonitis, where prognosis can be accurately predicted as early as 6 h post-insult based on the degree of myocardial depression (low stroke volume, high heart rate)[2]. This model offers a useful means of testing safety and efficacy.

## Objectives

To compare dose-related haemodynamic effects of esmolol at 6 hours in predicted survivors and non-survivors from faecal peritonitis.

## Methods

Instrumented male Wistar rats (350 ± 16 g) had sepsis induced with intraperitoneal injection of faecal slurry. Fluid resuscitation (10 ml/kg/h) was begun 2 h later. At 6 h, animals were divided into predicted survivors or non-survivors depending on a stroke volume cut-off of 0.20 ml. After an additional 10-ml/kg fluid bolus, esmolol was administered as a 500- µg/kg loading dose followed by an increasing stepwise infusion (50 to 200 µg/kg/min in 25- µg/kg/min increments 5 minutes apart). Heart rate, stroke volume and mean arterial pressure were recorded just prior to each dose increase. Repeated measures ANOVA and post-hoc Holm-Sidak test were used to seek statistically significant differences.

## Results

**Figure 1:**
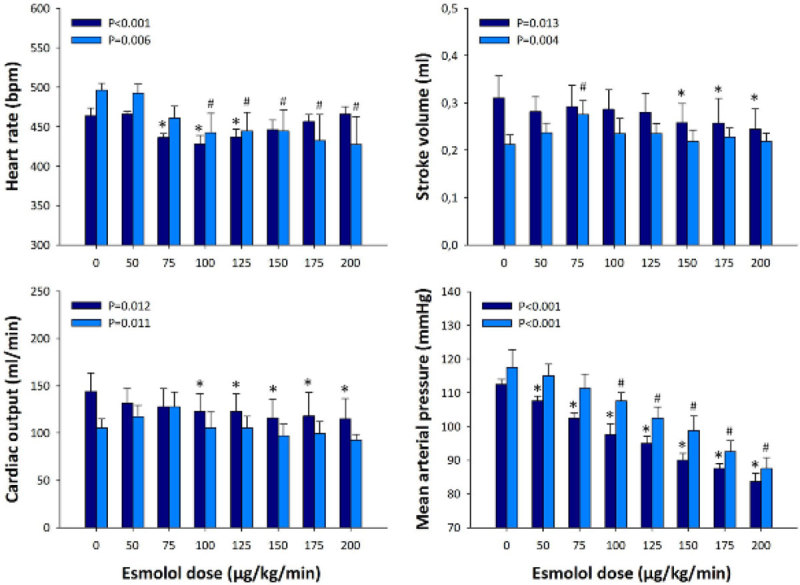
*p < 0.05 ***vs.*** **baseline (good prognosis group, dark blue bars, N = 4).** ^**#**^ **p < 0.05** ***vs.*** **baseline (poor prognosis group, light blue bars, N = 4).**

Baseline stroke volume at 6h was significantly lower in poor prognosis animals (0.27 ± 0.07 vs. 0.18 ± 0.02 ml, p < 0.05). Stroke volume increased with low dose esmolol in predicted non-survivors, and this offset the reduction in heart rate (Figure 1). Cardiac output was thus maintained in predicted non-survivors but fell significantly in predicted survivors. Mean BP fell in parallel in both groups, though significant changes were seen earlier in predicted survivors.

## Conclusions

Depending on their prognosis, septic rats show different haemodynamic responses to a short-term esmolol infusion at 6 h post-septic insult. Whether longer-term infusion is beneficial or harmful to these subgroups will be the subject of future study.

## Grant Acknowledgement

ESICM Basic Science Award, UK Intensive Care Society Young Investigator Award, NIHR.
